# A novel technique to retrieve cold‐welded implant abutment: A case series

**DOI:** 10.1002/ccr3.2375

**Published:** 2019-08-16

**Authors:** Mahnaz Arshad, Hossein Ali Mahgoli, Kamran Rasouli, Sina Refoua

**Affiliations:** ^1^ Prosthodontics Department, Dental Research Center, Dentistry Research Institute, School of Dentistry, International Campus Tehran University of Medical Sciences Tehran Iran; ^2^ Department of Prosthodontics, School of Dentistry Tehran University of Medical Sciences Tehran Iran; ^3^ School of Dentistry, International Campus Tehran University of Medical Sciences Tehran Iran; ^4^ Tehran University of Medical Sciences Tehran Iran

**Keywords:** abutment, implant, implant‐abutment connection

## Abstract

Abutment fracture is a complication of dental implant treatment. When an abutment breaks, the remaining part should be retrieved without damaging the implant hex. In many cases, the implant‐abutment connection is cold‐welded, which makes it difficult to remove the remaining part. The aim of the present study was to describe a simple technique to retrieve the remaining part of a broken abutment.

## 1 INTRODUCTION

Dental implant treatment is a predictable and reliable treatment option for the replacement of the lost teeth and dental rehabilitation in partially or fully edentulous patients. However, dental implants have some complications as well.^1^ Implant complications are categorized into two groups of mechanical and biological complications. According to Jung et al, peri‐implant mucosal lesions are the most common biological complications while the abutment or occlusal screw loosening, fracture of the implant body or prosthetic components, degradation of the luting cement, and fracture of the veneering are the most common mechanical complications.^2^

Fracture of implant components is rare with a prevalence rate of 0.4% to 2% over a 5‐year period. It more commonly occurs in the posterior region in partially edentulous patients^2^, ^3^, ^4^ and can be due to material flaw, poorly fitted prosthetic framework, acute trauma, screw loosening, and chronic mechanical and physiological overload.^4^, ^5^, ^6^, ^7^, ^8^

Cold welding refers to an increase in the loosening torque with respect to the tightening torque, which complicates the abutment retrieval.^9^ Based on a previous study, cold welding occurs as the result of bone debris and dried blood remaining on the surfaces of implant components such as the abutment screw and implant hex; the blood fibrin serves as a glue.^10^

The cold welding phenomenon at the implant/abutment connection may occur in two areas namely between the abutment screw and implant, and between the abutment and body of implant.

Abutment fracture can cause serious problems for both the clinician and patient.^10^, ^11^ The remaining part of a broken abutment within the implant prevents restoring the implant. In this situation, regardless of etiology, the remaining part must be removed without damaging the internal implant threads, which is a remarkably challenging task for clinicians. The condition is more complicated when the fracture is located at the internal hexagon joint of the abutments.^6^, ^7^, ^8^, ^12^

Various methods have been introduced for easy retrieval of the retained component without damaging the internal surface such as ultrasonic tool under a liquid coolant, dental extraction forceps, casting self‐made screwdriver, drilling instruments, submerging implants, or complete implant removal.^7^, ^10^

Search of the literature in PubMed and other databases revealed no universally acceptable technique for abutment retrieval. Although the aforementioned techniques may be helpful, they cannot completely fulfill the clinicians’ demands.

The aim of this case series was to describe a new technique to expedite the process of retrieval of a cold‐welded broken dental implant abutment.

## 2 MATERIALS AND METHODS

**Figure 1:**
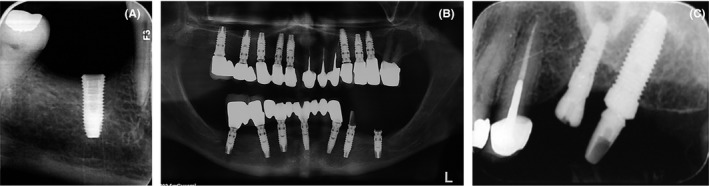
A, B and C; Broken implant abutments observable on radiographs

This clinical case series presents a new technique to retrieve cold‐welded dental implant abutments. A number of patients who were referred to our clinic with the chief complaint of fracture of implant‐supported fixed partial denture were evaluated. Clinical examination and taking patients’ medical and dental history revealed that some patients had a history of heavy bruxism (which was obvious considering the occlusal wearing noticed in their clinical intraoral examination) and insufficient number of implants. Periapical radiographs were obtained to assess the fractured abutment and the condition of fixture. The broken abutments were observed on radiographs (Figure 1).

**Figure 2:**
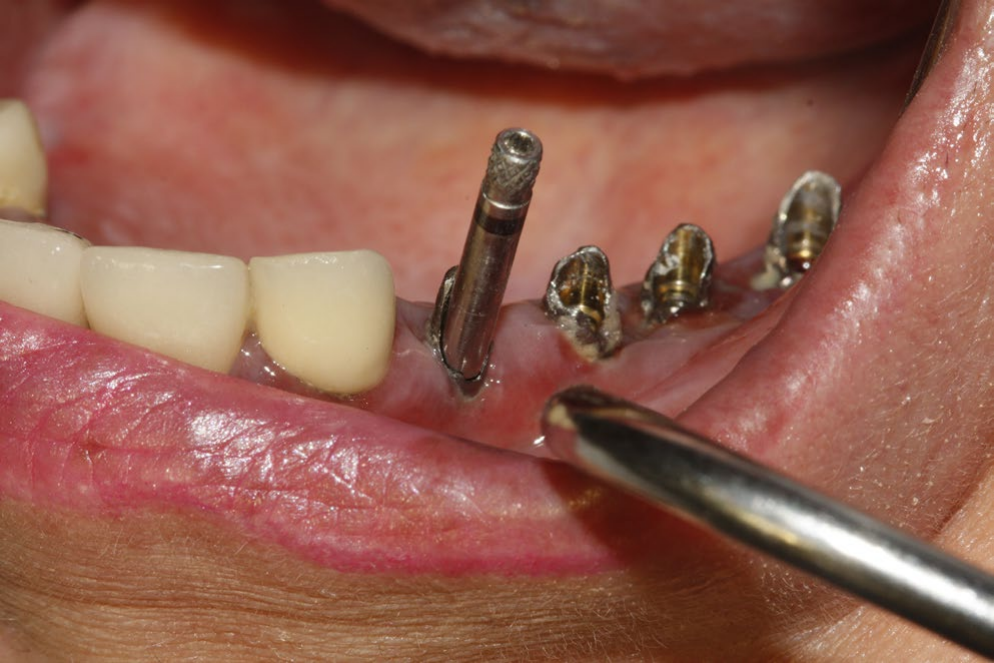
Screwing a long impression coping into the abutment

**Figure 3:**
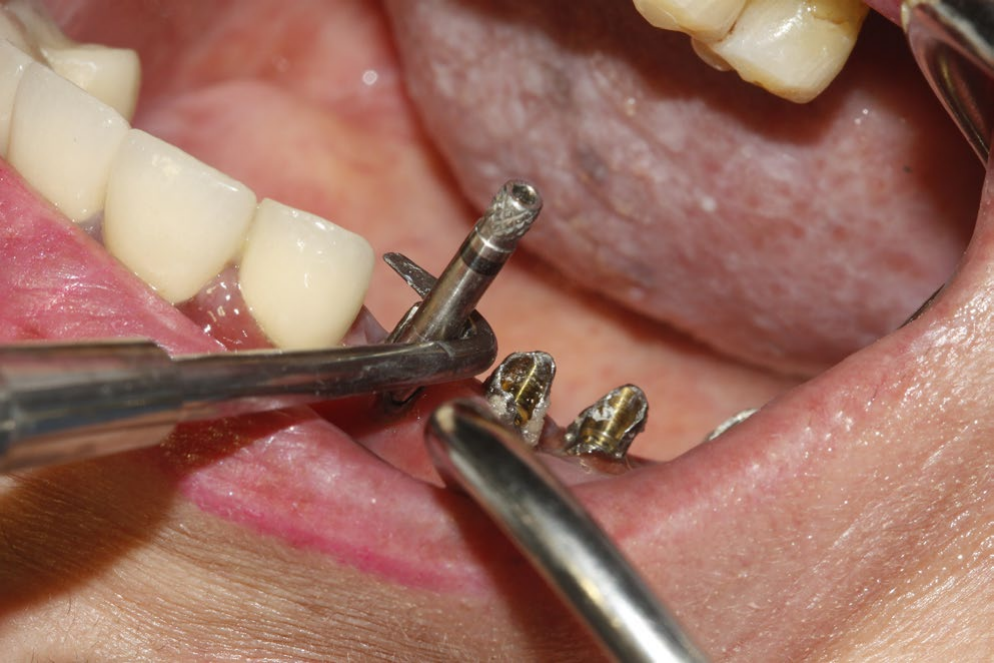
Multidirectional strokes with crown removal

**Figure 4:**
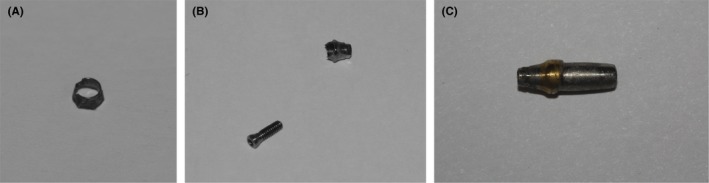
A, B and C; Retrieved abutment hex

In order to remove the remaining part of the abutments, the abutment screws were removed by a screwdriver. Dislodgment of the fractured abutments by the use of a hemostat or extraction forceps was not feasible since the abutments were cold‐welded and the fracture line was right at or slightly higher than the fixture‐abutment interface. Regarding the aforementioned limitations, the authors performed the following: First of all, the abutment screws were removed and then a long impression coping screw was placed on the abutments and screwed (Figure 2). Then, the arch crown hook was inclined horizontally to the screw and with applying few multidirectional strokes carried out in a very gentle manner, and the abutments were dislodged and easily removed. Glycerin was used as lubricant during the procedure (Figures 3 and 4).

## 3 DISCUSSION

Implant abutment fracture is a fairly rare complication in implant dentistry which can create an inconvenient situation for both the clinician and patient.^13^ It has been discussed that prevention is the best approach to avoid this complication. This can be achieved by following some specific principles such as placing an adequate number of implants with respect to the extent of the edentulous area, applying appropriate tightening torque as recommended by the manufacturer, applying a pretorque to ensure that implant components are fitted precisely, and removing the loosened screws instead of retightening them. In fact, regular follow‐ups play a key role to prevent screw loosening and fracture of implants.^12^

However, when abutment fracture occurs, the remaining part must be removed gently in order not to damage the internal threads of the implant. This could be extremely difficult for the clinicians since no specific method has been introduced for this purpose. Therefore, various innovative methods have been proposed by the clinicians.

Based on the literature, some factors affect the implant/abutment connection system including the connection type (internal connection versus the external connection), screw head design, screw material, screw diameter, preload, joint separating forces, and settling effect. The implant‐abutment interface connection can be internal connection or external connection. The internal connection has a much more important role in the stability of abutments and force distribution compared with the external connection.^14^

A screw joint between the implant and abutment is tightened by applying a torque to the screw abutment. The force generated within the screw by applying torque is known as the preload. Screw tightening produces tension in the screw, which causes elastic recovery and pulls the abutment and implant toward each other, creating a clamping force. Applying an adequate preload has some advantages such as lower micromotion of implant‐abutment screw interface, less frequency of screw loosening, improvement of fatigue resistance, and the locking of implant‐abutment connection.^14^

Permanent changes and plastic deformation occur as the result of a large preload applied to the material structure, and sometimes cold welding occurs in this condition. The recommended amount of torque for a preload to prevent this condition and ensure the safety of screw joint should be 75% of the total amount commonly applied for tightening.^13^

Cold welding is a rare mechanical complication of abutments. Nonetheless, it can be a serious problem and a cold‐welded abutment needs to be retrieved without damaging the internal implant thread. There are some case reports presenting different approaches and instruments for retrieval of cold‐welded abutment screws and healing abutments but there are no case reports to present an efficient approach for retrieval of cold‐welded abutments. In this paper, we presented a new technique for the first time for retrieval of a cold‐welded abutment which enabled successful retrieval of the abutment.

Some factors are important to prevent cold welding such as correct treatment planning, appropriate tightening torque, familiarity with various retrieval systems, and taking adequate precautionary measures before tightening of the cover screw.^10^ An ideal method for this purpose should meet the following criteria: (a) It must not be detrimental to the adjacent healthy tissues, (b) implant threads must remain intact, and (c) it should be applicable to all implant systems.

The technique described in this paper does not cause a temperature increase, unlike using ultrasonic or rotary instruments. Therefore, it is not as harmful as the aforementioned techniques for the healthy tissues. Also, it can be used for all dental implant systems while it does not damage the internal implant surface because the impression screw and the involved implant are from the same implant system. Furthermore, this technique is not costly or time‐consuming compared with other techniques such as fabrication of a custom guide tube or drilling the cover screws.

## 4 CONCLUSION

The technique introduced in this study is simple, cost‐effective, and efficient for both the patient and clinician and is applicable to all dental implant systems. However, more studies are needed to evaluate its reliability and generalizability.

## CONFLICT OF INTEREST

None declared.

## AUTHOR CONTRIBUTIONS

Mahnaz Arshad: provided idea, performed experimental design and hypothesis, wrote the manuscript, contributed substantially to discussion, and proofed the manuscript. Hoseinali Mahgoli: provided idea, performed hypothesis and experimental design, and contributed substantially to discussion. Kamran Rasouli: wrote the manuscript, contributed substantially to discussion, and submitted manuscript. Sina Refoua: contributed substantially to discussion and statistical analysis.
